# Supplementary material for: Transient expression of the neuropeptide galanin modulates peripheral‑to‑central connectivity in the somatosensory thalamus during whisker development in mice
Source: Nat Commun. 2024 Mar 29;15:2762. doi: 10.1038/s41467-024-47054-5 (PMC10980825; doi:10.1038/s41467-024-47054-5)
Supplement: Supplementary file 1 — Supplementary Information [file 41467_2024_47054_MOESM1_ESM.pdf]

Supplementary Information to the manuscript:

Transient expression of the neuropeptide galanin modulates peripheral-to-central connectivity in the somatosensory thalamus during whisker development in mice

Zsofia Hevesi, Joanne Bakker, Evgenii O. Tretiakov, Csaba Adori, Anika Raabgrund, Swapnali S. Barde, Martino Caramia, Thomas Krausgruber, Sabrina Ladstätter, Christoph Bock, Tomas Hökfelt[§] and Tibor Harkany[§]

[§]Correspondence should be addressed to either **Tomas Hökfelt** (Tomas.Hokfelt@ki.se) or **Tibor Harkany** (Tibor.Harkany@meduniwien.ac.at).

This file contains:

- 4 supplementary figures,
- 3 supplementary tables,
- legends to supplementary figures and tables.

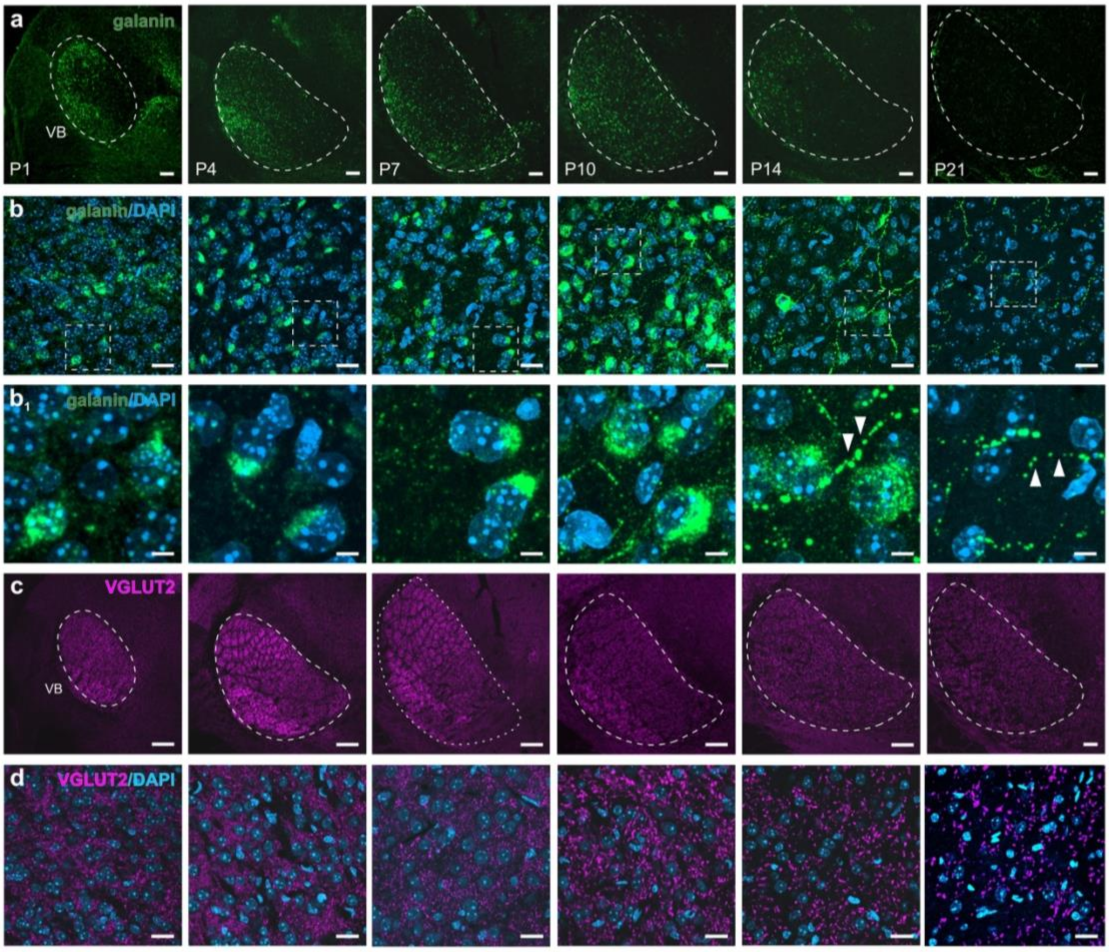

Supplementary Figure 1. Galanin and VGLUT2 expression in the ventrobasal thalamus during the early postnatal period. (**a**,**b**) Galanin was transiently expressed in cell bodies in the ventrobasal thalamus (VB) during P1-P14, peaking between P4-P10, with galanin accumulating in corticopetal projections from P10 onwards (**b_1,** *arrowheads*). (**c**) VGLUT2$^+$ terminals were already present at P1, with their organization into barreloids resolved by P4 and P7. (**d**) High-resolution images per age group are shown. Photomicrographs correspond to the numerical data in Figure 2 and are representatives of each group reported therein. *Scale bars* = 100 µm (a,c); 20 µm (b,d); 5 µm (b_1).

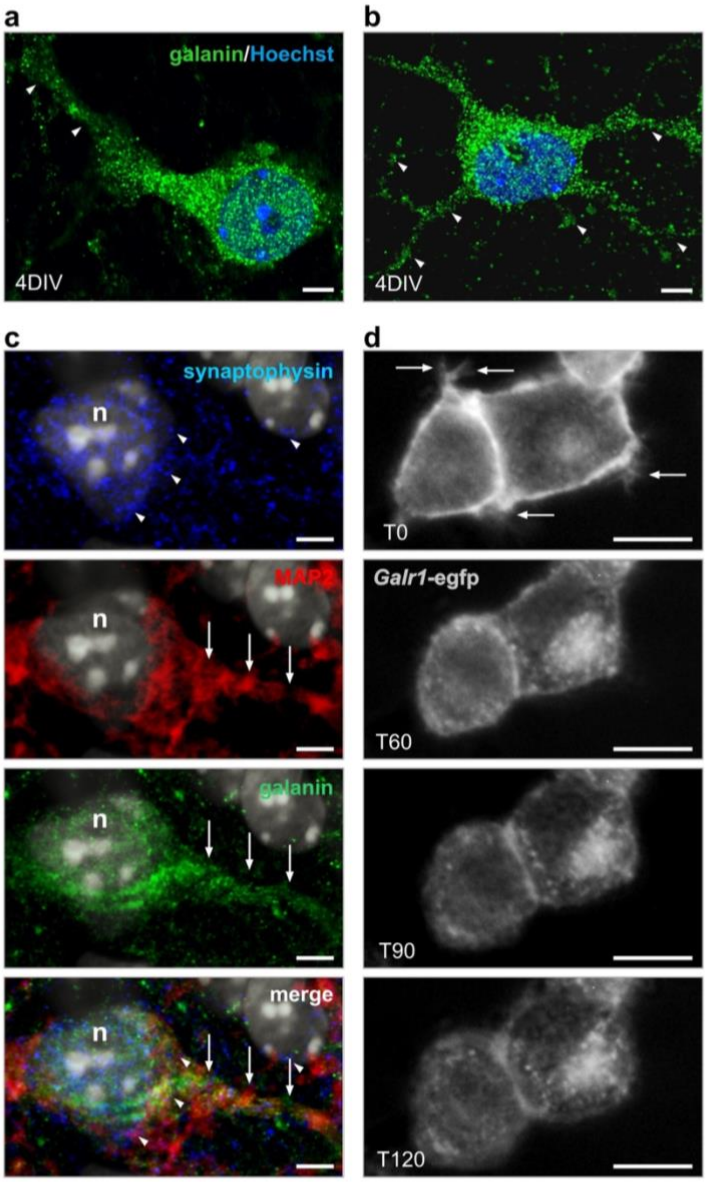

Supplementary Figure 2. Intracellular localization of galanin *in vivo* and *in vitro*. (**a**,**b**) High-resolution airyscan imaging (Zeiss) revealed dense galanin-like labeling in thalamic neurons. Galanin was broadly present in all neuronal compartments (*arrowheads*) when cell isolation was performed on P3, with cultures maintained for 4 days *in vitro* (4DIV). (**c**) Galanin also accumulated in dendrites labelled for microtubule-associated protein 2 (MAP2; *arrows*), and which apposed synaptophysin-1^{+} presynapses (*arrowheads*). Images were from P7 pups. Sections were counterstained by Hoechst 33,421, a nuclear dye ('n' labels the nucleus), which was color-coded in light grey. (**d**) PC12 cells stably expressing Gal_1R-EGFP chimeras were stimulated with galanin (100 nM)[46] and imaged at the time-points (T; in min) indicated. Experiments were performed in duplicate. *Arrows* point to Gal_1R-EGFP-containing filo- and lamellipodia, which rapidly retracted upon stimulation. *Scale bars* = 6 μm (**d**), 3 μm (**a-c**).

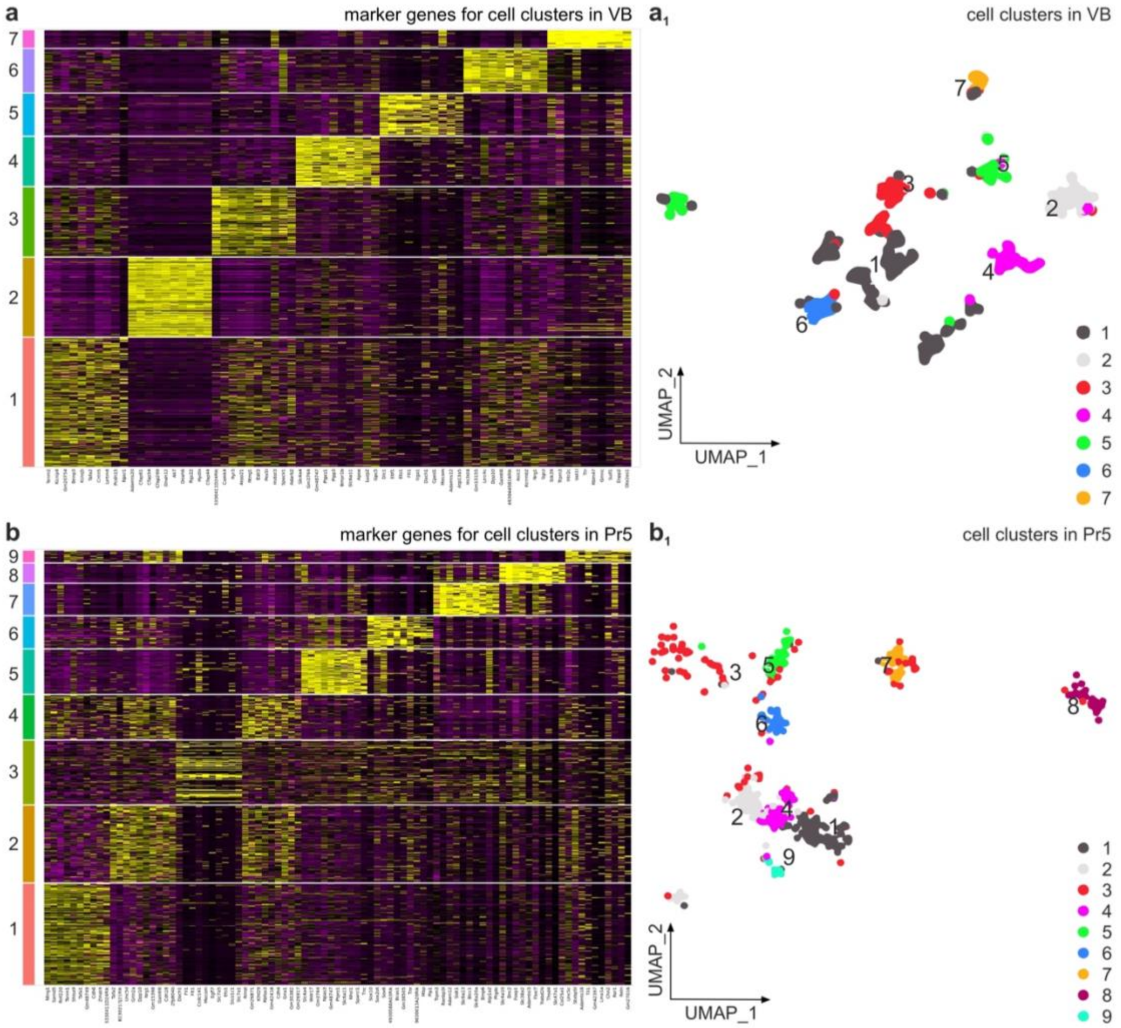

Supplementary Figure 3. Cellular diversity in the VB and the Pr5 on postnatal day 7. (**a**,**b**) Heat-map of associations between cell clusters and differentially expressed genes in the ventrobasal thalamus (VB, **a**) and the principal trigeminal sensory nucleus (Pr5, **b**). (**a$_1$**,**b$_1$**) UMAP representation of cellular clusters unmixed from both VB and Pr5.

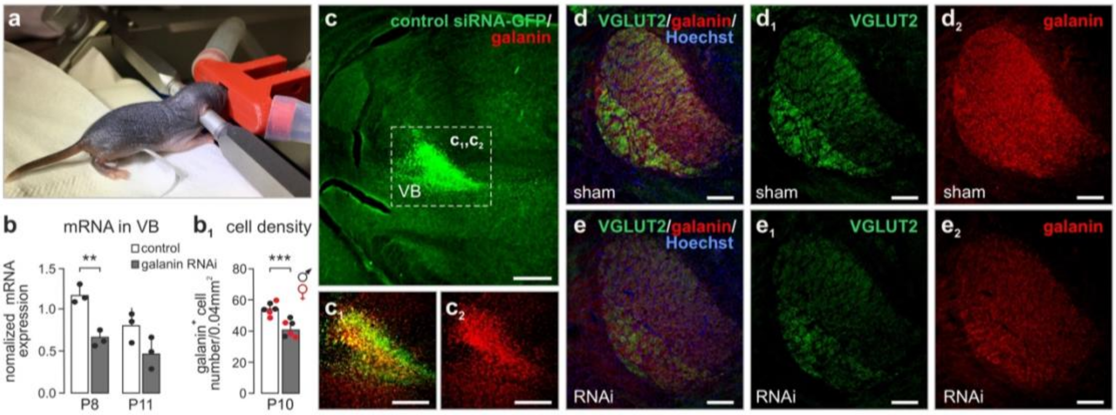

Supplementary Figure 4. ***In vivo*** **gene silencing decreased galanin expression in the ventrobasal thalamus (VB).** (**a**) A novel stereotaxic setup integrating gas anesthesia was used to inject AAV particles in infant mice[26]. (**b**) RNAi in the thalamus significantly reduced galanin levels measured 3 days (P8; n = 3 mice/group; p = 0.003; two-tailed unpaired Student's t-test) or 6 days (P11) after treatment. (**c**) Injection site in the VB visualized by GFP-tagged scrambled RNA (**c_1**), in overlap with galanin immunoreactivity (**c_2**) on P10. Both galanin expression (**b_1**; n = 6 mice/group; both sexes; p = 0.0004; two-tailed unpaired Student's t-test) and VGLUT2$^+$ synapse density (**d-e_2**) were found reduced in RNAi-injected infants (**e-e_2**) *vs*. sham-operated (**d-d_2**) at P10. Note, that these changes were specifically analyzed in the medial subdivision of the VB, which is the main relay of trigeminal whisker afferents originating from Pr5. Data in bar graphs were expressed as means ± s.e.m. Red and black data points denote female and male subjects, respectively. Images in (c)-(e_2) are representative for the animal groups used in quantitative morphometric analyses. *Scale bars* = 500 µm (**c-c_2**); 200µm (**d-e_2**).

Supplementary Table 1. Primary antibodies and other reagents used for immunohistochemistry, including tyramide signal amplification, and nuclear counterstaining

Marker	Host	Source	Dilution	Catalogue no.
Cre	Rabbit, pc[2]	Synaptic Systems	1:5,000, TSA	#257-003
DAPI	-	Thermo Scientific	1:1,000, IHC	#62248
Galanin	Rabbit, pc[2]	Peninsula Laboratories	1:2,000, IHC, IC	#T-4334
Galanin	Rabbit, pc[2]	Gift of E. Theodorsson, Linköping University	1:8,000, TSA	n/a
GFP	Chicken, pc[2]	Abcam	1:800, IHC	#13970
FITC-conjug. GFP	Goat, pc[2]	Abcam	1:1,000, IHC	#6662
Hoechst 33,342	-	Sigma	1:10,000, IHC, IC	#23491-52-3
MAP2	Guinea pig, pc[2]	Synaptic Systems	1:500, IHC	#188-004
NeuN	Mouse, mc[1]	Merck Millipore	1:1,000, IHC	#mab377
RFP	Rabbit, pc[2]	Rockland	1:1,500, IHC	#600-401-379
RFP	Guinea pig, pc[2]	Synaptic Systems	1:1,000, IHC	#390-005
Synaptophysin 1	Chicken, pc[2]	Synaptic Systems	1:500, IHC	#101006
VGLUT2	Guinea pig, pc[2]	Merck Millipore	1:1,500, IHC	#AB2251-1
VGLUT2	Guinea pig, pc[2]	Synaptic systems	1:500, IHC	#135-404

[1]monoclonal antibody,
[2]polyclonal antibody

Supplementary Table 2. Secondary antibodies for both indirect immunohistochemistry and tyramide signal amplification

Antibody	Species	Method	Dilution	Supplier
Anti-rabbit HRP	Swine	TSA	1:200	Dako, #P0217
Anti-chicken Cy2	Donkey	indirect	1:80	Jackson ImmunoResearch, #703-225-155
Anti-mouse FITC	Donkey	indirect	1:100	Jackson ImmunoResearch, #715-095-150
Anti-mouse AF647	Donkey	indirect	1:150	Jackson ImmunoResearch, #715-605-151
Anti-mouse Cy2	Donkey	indirect	1:150	Jackson ImmunoResearch, #715-225-151
Anti-rabbit Cy3	Donkey	indirect	1:150	Jackson ImmunoResearch, #711-165-152
Anti-guinea pig Cy3	Donkey	indirect	1:150	Jackson ImmunoResearch, #706-165-148
Anti-guinea pig AF647	Donkey	indirect	1:150	Jackson ImmunoResearch, #706-605-148

Supplementary Table 3. Primer sequences used for RT-qPCR

Target genes, accession number	Sequence (5´to 3´)	Reference
Gapdh<br>NM_008084	F: AACTTTGGCATTGTGGAAGG<br>R: ACACATTGGGGGTAGGAACA	Ref.[35]
Gal<br>NM_010253.4	F: AACAGCGCTGGCTACCTTCT<br>R: CTGTGAGGCCATGCTTGTCG	This study
