# Supplementary material for: Transient expression of the neuropeptide galanin modulates peripheral‑to‑central connectivity in the somatosensory thalamus during whisker development in mice
Source: Nat Commun. 2024 Mar 29;15:2762. doi: 10.1038/s41467-024-47054-5 (PMC10980825; doi:10.1038/s41467-024-47054-5)
Supplement: Supplementary file 3 — Inventory of Supplementary Information [file 41467_2024_47054_MOESM3_ESM.pdf]

Inventory of Supporting/Supplementary Information:

Transient expression of the neuropeptide galanin modulates peripheral-to-central connectivity in the somatosensory thalamus during whisker development in mice

Zsofia Hevesi, Joanne Bakker, Evgenii O. Tretiakov, Csaba Adori, Anika Raabgrund, Swapnali S. Barde, Martino Caramia, Thomas Krausgruber, Sabrina Ladstätter, Christoph Bock, Tomas Hökfelt[§] and Tibor Harkany[§]

[§]Correspondence should be addressed to either **Tomas Hökfelt** (Tomas.Hokfelt@ki.se) or **Tibor Harkany** (Tibor.Harkany@meduniwien.ac.at).

Supporting Information:

- Source data file,
- Author Checklist,
- Editorial Policy Checklist,
- Reporting Summary,
- Author list change form,
- Featured Image suggestion.

The Supplementary Information file contains:

- 4 supplementary figures,
- 3 supplementary tables,
- legends to supplementary figures and tables.
