# Supplementary material for: Transient expression of the neuropeptide galanin modulates peripheral‑to‑central connectivity in the somatosensory thalamus during whisker development in mice
Source: Nat Commun. 2024 Mar 29;15:2762. doi: 10.1038/s41467-024-47054-5 (PMC10980825; doi:10.1038/s41467-024-47054-5)
Supplement: Supplementary file 4 — Reporting Summary [file 41467_2024_47054_MOESM4_ESM.pdf]

nature portfolio

Corresponding author(s): Dr. Tibor HARKANY

Last updated by author(s): Feb 21, 2024

Reporting Summary

Nature Portfolio wishes to improve the reproducibility of the work that we publish. This form provides structure for consistency and transparency in reporting. For further information on Nature Portfolio policies, see our Editorial Policies and the Editorial Policy Checklist.

Statistics

For all statistical analyses, confirm that the following items are present in the figure legend, table legend, main text, or Methods section.

n/a	Confirmed	
☐	☒	The exact sample size (*n*) for each experimental group/condition, given as a discrete number and unit of measurement
☐	☒	A statement on whether measurements were taken from distinct samples or whether the same sample was measured repeatedly
☐	☒	The statistical test(s) used AND whether they are one- or two-sided<br>*Only common tests should be described solely by name; describe more complex techniques in the Methods section.*
☐	☒	A description of all covariates tested
☐	☒	A description of any assumptions or corrections, such as tests of normality and adjustment for multiple comparisons
☐	☒	A full description of the statistical parameters including central tendency (e.g. means) or other basic estimates (e.g. regression coefficient) AND variation (e.g. standard deviation) or associated estimates of uncertainty (e.g. confidence intervals)
☐	☒	For null hypothesis testing, the test statistic (e.g. *F*, *t*, *r*) with confidence intervals, effect sizes, degrees of freedom and *P* value noted<br>*Give P values as exact values whenever suitable.*
☒	☐	For Bayesian analysis, information on the choice of priors and Markov chain Monte Carlo settings
☒	☐	For hierarchical and complex designs, identification of the appropriate level for tests and full reporting of outcomes
☒	☐	Estimates of effect sizes (e.g. Cohen's *d*, Pearson's *r*), indicating how they were calculated

Our web collection on statistics for biologists contains articles on many of the points above.

Software and code

Policy information about availability of computer code

Data collection	1) Images were captured (and preprocessed with preserving linearity in Zen 2022 (Zeiss).<br>2) Lightsheet microscopy were captured and processed in the ImspectorTM software 1.0 (Abberior Instruments).<br>3) qPCR data were acquired by using the Bio-Rad CFX Manager software (version 3.1, Bio-Rad).<br>4) snRNA-seq data were acquired by Illumina commercial software (HCS v3.4.0, RRID:SCR_016386).<br>5) Behavioral data were collected (and processed) by using the Ethovision X15 software (Noldus).
Data analysis	1) Images were processed and analyzed in Imaris x64 9.0.2 (Bitplane) and/or Fiji 1.52e (GNU General Public Licence, https://imagej.net/Fiji). Cell counting was aided by the Cell Counter plug-in in ImageJ (1.49v; NIH) .<br>2) Cell Ranger (v7.1.0, RRID:SCR_017344), ZEN (Black, 2022, RRID:SCR_013672), ImageJ (v1.49, RRID:SCR_003070), GraphPad Prism (v8, RRID:SCR_002798) were used, all in commercially-available configurations without the introduction of custom-made codes.<br>We used existing R packages (RRID:SCR_001905) and Python modules (RRID:SCR_008394) as outlined in the on-line methods (https://harkany-lab.github.io/Hevesi_2023/methods.html). All versions are additionally available in a JSON file at https://raw.githubusercontent.com/Harkany-Lab/Hevesi_2023/main/output/methods/package-versions.json. Data availability: snRNA-seq data were deposited in NCBI Gene Expression Omnibus with accession number GSE230180. Code availability: The code developed and used in this report has been published at https://harkany-lab.github.io/Hevesi_2023/eda.html#Separate_analysis.<br>3) Behavioral data were analyzed in Ethivision X15 (Noldus).<br>4) All other data were analyzed in Microsoft Excel (v. 16.82) or Prism 8.0 (GraphPad).

For manuscripts utilizing custom algorithms or software that are central to the research but not yet described in published literature, software must be made available to editors and reviewers. We strongly encourage code deposition in a community repository (e.g. GitHub). See the Nature Portfolio guidelines for submitting code & software for further information.

Data

Policy information about availability of data

All manuscripts must include a data availability statement. This statement should provide the following information, where applicable:

- Accession codes, unique identifiers, or web links for publicly available datasets
- A description of any restrictions on data availability
- For clinical datasets or third party data, please ensure that the statement adheres to our policy

Data generated in this study are made available for download in both raw and processed forms from the NCBI Gene Expression Omnibus, with accession number: GSE230180. An archived version of the submitted code together with data to reproduce figures from snRNA-seq data is available on Figshare with DOI: 10.6084/m9.figshare.22665352.

Research involving human participants, their data, or biological material

Policy information about studies with human participants or human data. See also policy information about sex, gender (identity/presentation), and sexual orientation and race, ethnicity and racism.

Reporting on sex and gender	N/A
Reporting on race, ethnicity, or other socially relevant groupings	N/A
Population characteristics	N/A
Recruitment	N/A
Ethics oversight	N/A

Note that full information on the approval of the study protocol must also be provided in the manuscript.

Field-specific reporting

Please select the one below that is the best fit for your research. If you are not sure, read the appropriate sections before making your selection.

☒ Life sciences ☐ Behavioural & social sciences ☐ Ecological, evolutionary & environmental sciences

For a reference copy of the document with all sections, see nature.com/documents/nr-reporting-summary-flat.pdf

Life sciences study design

All studies must disclose on these points even when the disclosure is negative.

Sample size	The sample size has been chosen as specified in previous publications PMID: 25030704; PMID: 24469251.
Data exclusions	No data were excluded from the analysis.
Replication	The experiments reported here were minimally performed in duplicates (biological repeats in two (or more) independent experimental settings). All attempts of replication were successful.
Randomization	Experimental animals used in this study were randomly assigned to experimental groups, within and across litters. Likewise, treatments were randomized across the sexes. Experimental data were then processed both without (first step) and then with (second step) sex assignment. Since neither genetic background nor the experimental manipulations produce a persistent phenotypes that could be visible to an investigator prior to e.g. behavioral tests, biological replicates and randomization were successful and adequate.
Blinding	Experimenters were not blinded to the experimental conditions because control and treatment groups were simultaneously tested throughout (minimally n > 3/group at any given time), and processed automatically to prevent experimental bias. Thus, blinding was not considered as a factor of objectivity.

Reporting for specific materials, systems and methods

We require information from authors about some types of materials, experimental systems and methods used in many studies. Here, indicate whether each material, system or method listed is relevant to your study. If you are not sure if a list item applies to your research, read the appropriate section before selecting a response.

Materials & experimental systems

n/a	Involved in the study
☐	☒ Antibodies
☒	☐ Eukaryotic cell lines
☒	☐ Palaeontology and archaeology
☐	☒ Animals and other organisms
☒	☐ Clinical data
☒	☐ Dual use research of concern
☒	☐ Plants

Methods

n/a	Involved in the study
☒	☐ ChIP-seq
☒	☐ Flow cytometry
☒	☐ MRI-based neuroimaging

Antibodies

Antibodies used

Primary antibodies:
Anti-Cre, host: rabbit, puirified polyclonal antibody, Synaptic Systems, 1:5,000, #257-003 (lot# 257-003/4),
Anti-galanin, host: rabbit, polyclonal antibody, Peninsula Laboratories, 1:2,000, #T-4334 (batch of 02/03/1994),
Anti-galanin, host: rabbit, polyclonal antibody, gift of Dr. E. Theodorsson (Linköping University), 1:8,000,
Anti-GFP, host: chicken, polyclonal antibody, Abcam, 1:800, #13970 (lot# GR236651-5),
FITC-conjugated anti-GFP, host: goat, polyclonal antibody, Abcam, 1:1,000, #6662 (batch of 31/08/2020),
Anti-MAP2, host: guinea pig, polyclonal antibody, Synaptic Systems, 1:500, #188-004 (batch of 29/05/2018),
Anti-NeuN, host: mouse, monoclonal antibody, Merck Millipore, 1:1,000, #mab377 (lot# 21030466),
Anti-RFP, host: rabbit, polyclonal antibody, Rockland, 1:1,500, #600-401-379 (lot# 34944),
Anti-RFP, host: guinea pig, polyclonal antibody, Synaptic Systems, 1:1,000, #390-005 (batch of 05/09/2017)
Anti-synaptophysin 1, host: chicken, polyclonal antibody, Synaptic Systems, 1:500, #101006 (batch of 24/02/2016),
Anti-VGLUT2, host: guinea pig, polyclonal antibody, Merck Millipore ,1:1,500, #AB2251-1 (lot# 2983096),
Anti-VGLUT2 , host: guinea pig, polyclonal antibody, Synaptic systems, 1:500, #135-404 (batch of 09/02/2019).

Secondary antibodies:
HRP-conjugated swine anti-rabbit, Dako, 1:200, #P0217 (lot: 20069509),
FITC-conjugated AffiniPure donkey anti-mouse, Jackson ImmunoResearch, 1:100, #714-095-150 (lot: 87527)
Cy2-conjugated AffiniPure donkey anti-mouse, Jackson ImmunoResearch, 1:150, #715-225-151 (lot: 137363),
Cy2-conjugated AffiniPure donkey anti-chicken, Jackson ImmunoResearch, 1:80, #703-225-155 (lot: 134839),
Cy3-conjugated AffiniPure donkey anti-rabbit, Jackson ImmunoResearch, 1:150, #711-165-152 (lot: 159918),
Cy3-conjugated AffiniPure donkey anti-guinea pig, Jackson ImmunoResearch, 1:150, #706-165-148 (lot: 157540),
Alexa Fluor 647-conjugated AffiniPure donkey anti-mouse, Jackson ImmunoResearch, 1:150, #715-605-151 (lot: 135090),
Alexa Fluor 647-conjugated AffiniPure donkey anti-guinea pig, Jackson ImmunoResearch, 1:150, #706-605-148 (lot: 135631).

Validation

In the sequence as listed above for primary antibodies, from the 'Antibody Registry' with IDs (RRID) as follows:
AB_518348, AB_13970, AB_305635, AB_2298772, AB_2209751, AB_2665454

or with citations:
Kang M, Zhang Y, Kang HR, Kim S, Ma R, Yi Y, Lee S, Kim Y, Li H, Jin C, Lee D, et al. CYFIP2 p.Arg87Cys Causes Neurological Defects and Degradation of CYFIP2. Annals of neurology (2023) 931: 155-163.
Asencor AI, Dvoryanchikov G, Makhoul V, Tsoulfas P, Chaudhari N. Selectively Imaging Cranial Sensory Ganglion Neurons Using AAV-PHP.S. eNeuro (2022) 93: 0373-21.2022.
Cheret C, Ganzella M, Preobraschenski J, Jahn R, Ahnert-Hilger G. Vesicular Glutamate Transporters (SLCA17 A6, 7, 8) Control Synaptic Phosphate Levels. Cell reports (2021) 342: 108623.

Animals and other research organisms

Policy information about studies involving animals; ARRIVE guidelines recommended for reporting animal research, and Sex and Gender in Research

Laboratory animals

For behavioral, imaging, neuroanatomy, and biochemical experiments, mice of both sexes were used, including the following strains and their eventual crosses: C57Bl6/N (as wild-type, local breeding); B6.Cg-Gt(ROSA)26Sortm14(CAG-tdTomato)Hze/J (The Jackson Laboratory #007914) "Ai14"; Gal(BAC)-Cre mice from GENSAT (line Ki87).Mice were kept under standard conditions of husbandry, with a 12h/12h light cycle and 55% humidity. Care was taken to minimize the number and the suffering of the mice used. Transgenic mice were backcrossed for multiple generations onto the C57BL/6N background. Gal-Cre mice were bred and crossed heterozygously. Infant mice were kept in their original litters, and separated from the lactating female only briefly (for the period of behavioral tests) to avoid any bias due to separation (stress, thermoregulatory changes etc.). Litters were of quasi-equivalent sizes for all strains and thus not trimmed.

Primary neuronal cultures were prepared from either P0 or P3 C57BL/6JRj mouse brains.

Wild animals

No wild animal was used.

Reporting on sex

Males and females were used in this study. Sex was specified by color coding in each relevant figure/figure panel.

Field-collected samples	No filed-collection took place.
Ethics oversight	Experimental procedures on mice conformed to the 2010/63/EU directive and were approved by the Austrian Ministry of Education, Science and Research (66.009/0145-WF/II/3b/2014 and 66.009/0277-WF/V3b/2017). All procedures were planned to reduce suffering, as well as animal numbers.

Note that full information on the approval of the study protocol must also be provided in the manuscript.

Plants

Seed stocks	N/A
Novel plant genotypes	N/A
Authentication	N/A
